# Cost-Effective Filter Materials Coated with Silver Nanoparticles for the Removal of Pathogenic Bacteria in Groundwater

**DOI:** 10.3390/ijerph9010244

**Published:** 2012-01-18

**Authors:** Lizzy Mpenyana-Monyatsi, Nomcebo H. Mthombeni, Maurice S. Onyango, Maggy N. B. Momba

**Affiliations:** 1 Department of Environmental, Water and Earth Sciences, Tshwane University of Technology, Arcadia Campus, Private Bag X680, Pretoria 0001, South Africa; Email: monyatsil@tut.ac.za; 2 Department of Chemical Engineering, Tshwane University of Technology, Arcadia Campus, Private Bag X680, Pretoria 0001, South Africa; Email: nomceboH@tut.ac.za (N.H.M.); onyangoms@tut.ac.za (M.S.O.)

**Keywords:** silver nanoparticles, filter systems, bacterial removal, water disinfection, groundwater

## Abstract

The contamination of groundwater sources by pathogenic bacteria poses a public health concern to communities who depend totally on this water supply. In the present study, potentially low-cost filter materials coated with silver nanoparticles were developed for the disinfection of groundwater. Silver nanoparticles were deposited on zeolite, sand, fibreglass, anion and cation resin substrates in various concentrations (0.01 mM, 0.03 mM, 0.05 mM and 0.1 mM) of AgNO_3_. These substrates were characterised by SEM, EDS, TEM, particle size distribution and XRD analyses. In the first phase, the five substrates coated with various concentrations of AgNO_3_ were tested against *E. coli* spiked in synthetic water to determine the best loading concentration that could remove pathogenic bacteria completely from test water. The results revealed that all filters were able to decrease the concentration of *E. coli* from synthetic water, with a higher removal efficiency achieved at 0.1 mM (21–100%) and a lower efficiency at 0.01 mM (7–50%) concentrations. The cation resin-silver nanoparticle filter was found to remove this pathogenic bacterium at the highest rate, namely 100%. In the second phase, only the best performing concentration of 0.1 mM was considered and tested against presumptive *E. coli*, *S. typhimurium, S. dysenteriae* and *V. cholerae* from groundwater. The results revealed the highest bacteria removal efficiency by the Ag/cation resin filter with complete (100%) removal of all targeted bacteria and the lowest by the Ag/zeolite filter with an 8% to 67% removal rate. This study therefore suggests that the filter system with Ag/cation resin substrate can be used as a potential alternative cost-effective filter for the disinfection of groundwater and production of safe drinking water.

## 1. Introduction

The World Health Organisation (WHO) has indicated that approximately 1.8 million deaths and 61.9 million disability-adjusted life years (DALYs) worldwide are attributable to unsafe water, sanitation and poor hygiene. An estimated 99.8% of such deaths occur in developing countries, with children ranking (90%) as the first victims [1]. Consumption of groundwater and surface water sources contaminated with pathogenic bacteria such as *Escherichia coli* O157:H7, *Salmonella typhimurium*, *Shigella dysenteriae* and *Vibrio cholera* continues to be one the major causes of diarrheal diseases and gastrointestinal infections [2,3,4,5,6]. This implies that safe drinking water plays a significant role in human health and well-being. 

In 2002, the United Nation Millennium Development Goals (MDG) firmly established the issue of water and sanitation on the global agenda. The vision of the MDG is to halve the number of people without access to safe drinking water and sanitation by 2015 [7]. Although tremendous progress has been made to date, the 2010 updated report by the WHO and the United Nations Children’s Fund (UNICEF) has indicated that 884 million people in the world still lack access to drinking water from improved sources. 

The provision of safe drinking water is currently a high priority for the South African government, one of the signatories of the MDG. The percentage of households with access to water infrastructure above or equal to the Reconstruction and Development Programme standard increased from 61.7% in 1994 to 91.8% in March 2009. Based on these data, it is estimated that 93% of the population had access to an improved drinking-water supply in 2010 [8]. In most of the cases, non-improved drinking water supplies are currently found in rural communities that are widely dispersed and informal peri-urban communities that are continuously expanding. It is therefore difficult to implement centralised systems such as piped systems, which not only require substantial financial support, but also highly skilled personnel to manage and maintain them. The implementation of decentralised systems is therefore needed to provide these rural communities with safe drinking-water sources. 

Cost-effective filter materials coated with silver nanoparticles is an alternative technology that could assist the developing countries in meeting the MDG, and South Africa, in particular, in providing a safe drinking-water supply to all scatted rural areas and informal settlements. Silver ion (Ag^+^) has long been known as a potential antimicrobial agent and is used in wound dressings to prevent infections in burn patients, to blindness in newborns, for severe chronic osteomyelitis and urinary infection, to control *Legionella* bacteria in hospitals and to improve the performance of drinking-water filters [9,10,11,12]. It can bind to bacterial cells and enzymes (proteins) at multiple sites, damaging them and preventing them from performing their functions and result, to cells death through penetration at specific bacterial DNA and RNA [9,13,14]. 

Silver in the form of nanoparticles that release silver ions more effectively has a better bactericidal activity due to its high surface-area-to-volume ratio [15,16]. Recent studies have shown that distinctively prepared silver nanoparticles display good antibacterial activity [17,18]. As a result, researchers have considered silver nanoparticles for drinking-water treatment due to its strong and broad spectrum of antimicrobial activities [19,20,21]. With the advancement of material development, silver nanoparticles can be easily deposited on solid materials for the deactivation of microorganisms in water treatment [22]. In the case of drinking-water treatment, various forms of silver nanoparticles coated on materials/substrates have been used. These include Ag/sand [23], Ag/zeolite [17] and Ag/fibreglass [24]. Sand filtrations have been used in water purification to control microbiological contamination for over 150 years [25]. Sand filters are a less expensive, more effective method of water treatment, can be self-constructed and may be constructed by using local skills. Natural zeolites as cation exchangers in water treatment have increased due to their availability, low cost, high surface area and sorptive capacity, negative surface charge, chemical inertness and low or null toxicity for human [26,27]. Most kinds of fibreglass are used for thermal and acoustic insulation in building construction, shipbuilding and filtration applications. Fibreglass-reinforced plastics (FRPs) have been used for various types of process equipment in the chemical industry, pulp and paper industry, power and mining industries, municipal sewer treatment and water treatment, as well as many other associated industries handling corrosive equipment [28]. A number of investigations have been carried out on the use of resins containing silver/silver nanoparticles for oral and dental applications [29,30]. Resins are used in ion exchange and constitute a very powerful technology for removing impurities from water and other solutions. There is no health risk with resins, as many industries use resins for multiple purposes (nuclear and thermal power stations, semiconductors, computers), including dental and pharmaceutical applications and drinking-water treatment for the removal of toxic contaminants [31,32]. 

Even though a number of studies have been conducted on bacterial removal with Ag/zeolite, Ag/sand, Ag/fibreglass and Ag/resin nanoparticle substrates, there is no information on comparative studies related to the use of these technologies for the removal of pathogenic bacteria from drinking-water sources. This study therefore concentrated on the development of these substrates modified with silver nanoparticles and compared their effectiveness in removing pathogenic bacteria (*Escherichia coli*, *Vibrio cholerae*, *Shigella dysenterae and Salmonella typhimurium*) from polluted groundwater sources. Our main intention was to find the alternative cost-effective technology with the best concentration of silver nanoparticles loaded on the substrates, which could completely remove pathogenic bacteria from test water and result in the production of safe drinking water for rural communities. 

## 2. Experimental Methodology

### 2.1. Preparation of Substrates

Locally available materials for silver deposition were utilised in the present study. Silver was coated on natural zeolite, sand, fibreglass, anion resin and cation resin. Natural clinoptilolite zeolite purchased from Ajax Industries CC (Cape Town, South Africa) was conditioned in a 500 mL solution of 2 M NaCl (Merck, South Africa), followed by stirring at room temperature (between 20 and 25 °C) for 36 h. The solid-liquid mixture was separated by centrifugation at 3,000 rpm for 15 min. Liquids were discarded and the solids were washed with deionised water three times, and then oven dried at 105 °C for 8 h. Silica sand purchased from Eggo Sand (Pty) Ltd (Pretoria, South Africa) was submitted to a cleaning process by stirring 200 g of sand in a litre of 30% nitric acid (Merck, South Africa) solution with a reciprocating shaker at 210 rpm at room temperature for 24 h. The sand was allowed to settle and separated from the solution by decantation, and thereafter rinsed three times with deionised water and oven dried at 105 °C for 24 h. Fibreglass chopped-strand mat was purchased from Collins Fibreglass Plastics (Johannesburg, South Africa) and cleaned by immersion in an ultrasonic bath containing isopropanol (Sigma, South Africa) for 2 h. The substrate was rinsed three times with deionised water and oven dried at 105 °C for 24 h.

### 2.2. Synthesis of Silver Nanoparticle-Coated Substrates

#### 2.2.1. Coating of Zeolite, Sand and Fibreglass Substrates

Silver nitrate (AgNO_3_) (Merck, South Africa) stock solution (1 mM) was prepared by adding 169.87 mg of silver nitrate to a litre of deionised water. Thereafter concentrations of 0.01, 0.03, 0.05 and 0.1 mM silver nitrate (250 mL) were prepared by diluting silver stock solution. The substrates (20 g) were separately immersed in aqueous solution containing concentrations of 0.01, 0.03, 0.05 and 0.1 mM silver nitrate for 24 h. They were incubated in a thermostatic shaker at a speed of 250 rpm in the dark at room temperature for 24 h. Substrates containing silver were separated from the mixture by centrifugation at 3,000 rpm for 15 min and washed with deionised water, and then oven dried for 24 h at 105 °C. For the reduction of silver ions to silver nanoparticles, the substrates containing silver were heated in an N_2_ furnace (Lenntech, South Africa) at a flow rate of 400 mL/min for 1 h at 120 °C and the furnace was ramped up to 350 °C for 3 h. 

#### 2.2.2. Coating of Anion Resin Beads Substrate

Amberlite-IRA-458 anion exchange resin (in chloride form) was purchased from Lenntech (Johannesburg, South Africa). Silver was coated on the anion resin beads using the method previously described by [33]. Briefly, the silver nanoparticle-resins were prepared following a two-step procedure. A known amount (20 g) of anion exchange resin was used. Firstly, 30 mL of 1 M HCl (Merck, South Africa) solution were added dropwise to 200 mL stirred, freshly prepared aqueous solution of concentrations of 0.01, 0.03, 0.05 and 0.1 mM AgNO_3_ to form white precipitates of silver chloride. The precipitates were washed three times with deionised water to remove HNO_3_ and dried in a water bath at 65 °C for 2 h. The silver precursor [AgCl_2_]^¯^ complex was prepared by dissolving 0.3 g of solid AgCl in a concentrated HCl solution and the mixture was placed in an ultrasonic bath for dissolution. Secondly, the silver precursor ions were allowed to exchange with Cl^¯^ ions of the neat chloride form of anion-exchange resin beads (R^+^Cl^¯^) and the mixture was kept overnight. The resin beads, on which silver precursor ions were immobilised, were washed three times with water to drain out the liberated HCl and un-exchanged [AgCl_2_]^¯^ and then reduced with a freshly prepared ice-cold aqueous solution of 0.01 M sodium borohydride. The prepared shining reddish-black silver-coated beads [R(Ag)^0^]^+^Cl¯ were washed thoroughly with deionised water and dried at room temperature in a vacuum. 

#### 2.2.3. Coating of Cation Resin Beads Substrate

The methods described by [34] were also used for the coating of cation resin. The silver amine complex, [Ag(NH_3_)_2_]^+^, was prepared by adding 10 mL of 25% ammonia solution dropwise to each of 200 mL aqueous solution of concentrations of 0.01, 0.03, 0.05 and 0.1 mM AgNO_3_. A known amount (20 g) of cation exchange resin (R^–^H^+^) was added to each of these mixtures, followed by a mixing process for 3 h using a magnetic stirrer. The resin silver amine moiety [R–Ag(NH_3_)_2_] was washed three times with deionised water and heated in an oven at 150 °C for 1 h. The yellow colour of the resin beads was transformed to black due to the formation of resin silver oxide composite [R(Ag_2_O)]¯H^+^. Then, this complex was reduced with an aqueous solution of freshly prepared 0.01 M sodium borohydride to form silver nanoparticle-coated resin beads [R(Ag^0^)]¯H^+^, with a white colour. The silver nanoparticle-resin beads were washed three times with deionised water and finally dried on a water bath at 65 °C for 2 h to obtain dry silver-coated resin beads. 

### 2.3. Characterisation of Substrates Coated with Silver Nanoparticles

The surface morphology of the silver nanoparticles-coated substrates was examined with a scanning electron microscope (SEM) (JEOL JSM-5800LV, JEOL Ltd, Tokyo, Japan) coupled with energy-dispersive spectroscopy (EDS) to confirm the chemical content on the substrates. Transmission electron microscope (TEM) analysis was performed with a JEOL 2100F (JEOL Ltd, Tokyo, Japan) that operated at 100 kV to examine the morphology and particle size distribution of the silver nanoparticle substrates. X-ray diffraction (XRD) was used to determine the crystal phase of the substrates. The patterns of the silver nanoparticles were recorded with a Bruker D8 Advance using Cu Kα radiation with 1.5416 Å wavelengths. The structure of the silver on the substrates was studied by scanning the media in 2θ ranges from 30 to 80 °C in a continuous scan mode. The crystallite size of the silver substrates was determined from X-ray line broadening using the Debye-Scherrer equation as follows: 


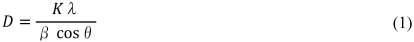


where D = Crystallite size, A (Angstroms), K = Crystallite-shape factor = 0.9, λ = X-ray wavelength, 1.5416 Å for CuKα, θ = Observed peak angle, degree, β = X-ray diffraction broadening, radian.

### 2.4. Production of Combined Substrate-Silver Nanoparticle Filter Systems

**Figure 1:**
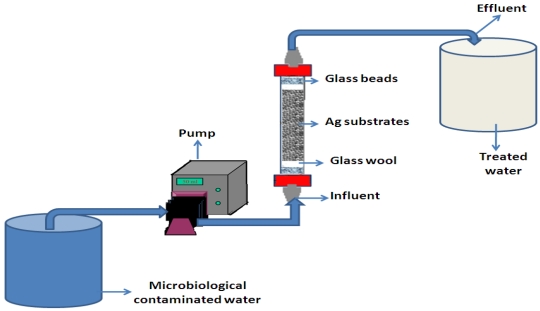
Schematic diagram of laboratory-scale setup to evaluate the antibacterial efficiency of Ag nanoparticle-coated substrates.

The filter systems consisted of a polyvinyl chloride (PVC) column of 2 cm diameter and 20 cm length (Figure 1). Each column was packed with one type of the substrate coated with silver nanoparticles at a depth of 10 cm. With reference to various substrates (sand, zeolite, fibreglass, anion resin and cation resin), five filters were used during the study period. Glass beads (2 cm) and glass wool (2 cm) were placed in the upper and bottom ends of each column. Glass beads were positioned to prevent the substrates from pilling up at one end. A 10 L bucket served as storage container for contaminated influent water, which was fed into the filter system with a 1 m length of 8 mm diameter latex tubing connected to a Rainin Dynamax peristaltic pump (Rainin Instrument Co., Woburn, MA, USA). The effluent sample (treated water) was collected at the top of the filter with a 1 m length of 8 mm diameter latex tubing into a 10 L bucket container. Figure 1 illustrates the schematic diagram of a laboratory-scale setup with an example of a combined substrate-Ag nanoparticle filter system.

### 2.5. Testing the Efficiency of Filter Systems in Removing Pathogenic Bacteria

The performance of the combined substrates-silver nanoparticle filter systems in removing pathogenic bacteria was studied in two phases. In the first phase, the five substrates coated with various concentrations of AgNO_3_ were tested against *Escherichia coli* (ATCC 43895) spiked in synthetic water samples. The main objective of this part of the study was to determine the best loading concentration of AgNO_3 _that could result in the total removal of pathogenic bacteria from a test water source. In the second phase, the performance of the five combined substrates-silver nanoparticle filter systems was tested using groundwater, and only the best concentration of silver nanoparticles loaded on the substrates was investigated against the four different pathogenic bacteria-*E. coli*, *S. typhimurium*, *S. dysenteriae* and *V. cholerae*. In cases where these pathogens were not present in the intake groundwater source, the water was spiked with the pathogens. This was mainly done to evaluate the efficiency of each filter in reaching the allowable recommended limits set by the *South African National Standards* for domestic use [35,36]. The bacterial removal efficiency was obtained by comparing the concentrations (Log_10_ cfu/100 mL) of target organisms before and after treatment. In each series of the experimental study, a control filter constituted of the substrate without silver nanoparticles was included. The experimental study for each combined substrates-silver nanoparticle filter system was performed in three different trials.

#### 2.5.1. Preparation of Bacterial Stock Suspensions

The microbial strains used in the experimental study included *Escherichia coli* (ATCC 43895) and *Salmonella typhimurium* (ATCC 14028) obtained from the American Type Culture Collection (Rockville, MD, USA), *Vibrio cholera* and *Shigella dysenteriae* obtained from the Council for Scientific and Industrial Research (CSIR, Pretoria, South Africa) bacterial stock cultures. Prior to use, these bacterial strains were confirmed by cultural tests using selective agar medium (Chromocult agar for *E. coli*, XLD agar for *S. typhimurium* and *S. dysenteriae*, TCBS agar for *V. cholera*) according to the Standard Method [37]. One loop full of each bacterial culture was separately inoculated in 100 mL sterile nutrient broth (Merck, South Africa) medium and incubated aerobically at 37 °C in a shaking incubator (Scientific Model 353, Lasec, South Africa) at 120 rpm for 24 h. The bacteria were harvested by centrifugation at 4,000 rpm for 10 min and the pellet was washed twice with 50 mL of sterile 0.01 M phosphate-buffered saline (PBS, pH 7.2). 

The stock suspensions of *E. coli*, *S. typhimurium*, *S. dysenteriae* and *V. cholera* were prepared by re-suspending the final pellets in 10 mL of 0.01 M PBS solution. The initial concentrations of bacterial cells harvested were determined with the spread-plate technique, after serial dilution of each culture in sterile saline solution (0.9% w/v NaCl). The plates were incubated at 37 °C for 24 h. The resulting colonies were counted and expressed as cfu/mL. 

#### 2.5.2. Preparation of Synthetic Contaminated Water

For each filter system, an aliquot of the stock suspension of *E. coli* (ATCC 43895) corresponding to 6 log cfu/100 mL was inoculated into 10 L final volumes of sterile saline water (8.5% NaCl). The spiked water samples were shaken vigorously several times and 1 mL of this water source was used to determine the initial concentration of the target organism before passing the remaining contaminated water through the filter systems. 

#### 2.5.3. Collection and Analysis of the Quality of Groundwater Samples

Groundwater samples were collected from a borehole at Delmas (A7) in the Mpumalanga Province of South Africa. The study was conducted between June and July 2010 and the water samples were collected three times during this period. It is important to note that, during the study period, this groundwater supply was used by the community without prior treatment. The water samples were collected in sterile 50 L plastic buckets. Samples were also collected in sterile 1 L glass bottles in order to detect and enumerate the initial concentrations of the target bacteria and selected physicochemical parameters before treatment. The samples were transported to the laboratory and the quality of the water was determined for microbiological contamination and selected physicochemical parameters within 6 h [37]. 

*Escherichia coli*, *S. typhimurium*, *S. dysenteriae* and *V. cholerae* were detected and enumerated from groundwater samples according to Standard Methods [37]. As mentioned above, in cases where these organisms were not detected in groundwater samples, they were spiked with 10^2^ cfu/mL stock suspension in these test water sources using the same method as described for synthetic water samples. All the tests were conducted in aseptic conditions. 

The pH and the turbidity were measured on site using a pH meter (Metrohm Co. Model 713) and a microprocessor turbidity meter (Eutech Instrument Turbidimeter TN-100), respectively. Nitrates and fluoride concentrations were determined in the laboratory using the Spectroquant Nova 400 manual water analyser (Merck) and photometric test kits (Merck), while the concentrations of magnesium, calcium in the water sample were determined by atomic absorption spectrophotometry (SpectrAA 220FS), according to Standard Methods [37]. 

#### 2.5.4. Operating Conditions and Testing the Performance of the Filter Systems

Before the start of each run, the packed columns were pumped upward with sterile deionised water to adjust and achieve a steady-state flow condition. Thereafter, each type of test water source contaminated with enteric pathogenic bacteria was pumped continuously through the column in the up-flow mode with a Rainin Dynamax peristaltic pump (Rainin Instrument Co.) that operated at a flow rate of 0.12 L/h. Treated water samples were collected in sterile conical flasks at 10-minute intervals at a volume of 50 mL. Prior to use, 1 mL 15% sodium thiosulphate was added to the flasks to stop further disinfection of drinking water before determining the bacterial concentrations. The bacterial concentrations were determined by serial dilution in sterile saline solution and plated on selective medium by using the spread-plate technique according to Standard Methods [37].

#### 2.5.5. Elution of Silver Ions from Silver Nanoparticles Substrates Filter Systems

The degree of elution of silver from each combined substrates-silver nanoparticle filter system used in the first phase with spiked synthetic water was measured. The silver content in the treated water samples was determined with atomic absorption spectroscopy (AAS) by using a Spectra AA-220FFS (Varian Medical Systems, Inc., Palo Alto, CA, USA).

### 2.6. Statistical Analysis

All data were analysed statistically using the SPSS computer software version 11.0. Testing of significance was carried out using the one-way analysis of variance (ANOVA) at a 95% confidence interval. Comparisons were made between the treatment means of each filter system to determine if there were significant differences in treatments. 

## 3. Results

### 3.1. Characterisation of Silver Nanoparticles Coated on Substrates

**Figure 2:**
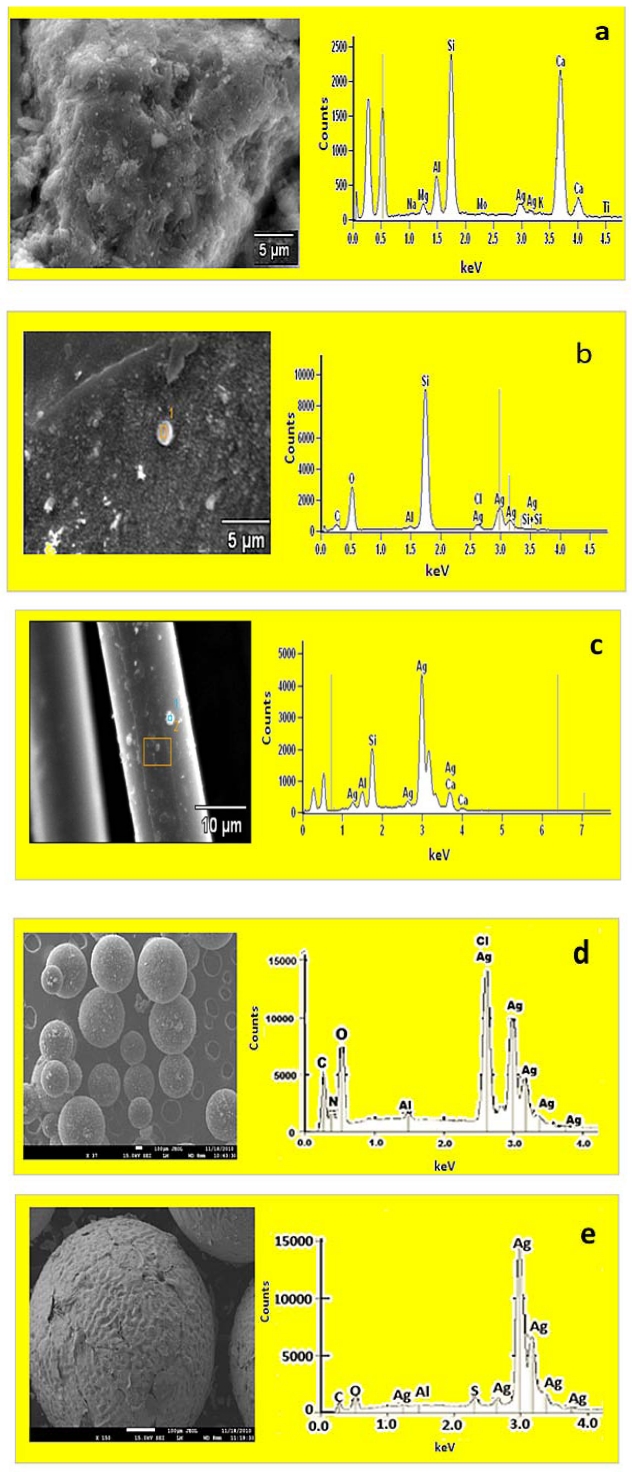
SEM image and EDS spectrum of (**a**) zeolite, (**b**) sand, (**c**) fibreglass, (**d**) anion resin, and (**e**) cation resin coated with silver nanoparticles.

Figure 2 shows the morphologies and the elemental composition of the silver nanoparticles coated on substrates (Ag/zeolite, Ag/sand, Ag/fibreglass, Ag/anion resin, Ag/cation resin) examined with SEM micrograph and EDS analyses. The silver nanoparticles were noticed as small white spherical particles on the surface of the sand, fibreglass and anion resin. In cation resin, the silver nanoparticle coating completely covered the resin bead and the film revealed a homogeneous rough surface. The structure of the silver nanoparticles in zeolite was complex due to sample charging. Zeolite sample charging could indicate a low electrical conductivity of the medium even though carbon coating had been applied to avoid charging. The EDS spectra also confirmed the elemental composition of silver nanoparticles by the presence of Ag peaks in the synthesised substrates. In the spectra, two peaks of Ag located between 3.0 kV and 3.5 kV were observed in zeolite, four peaks for sand located between 2.5 kV and 3.5, four peaks for fibreglass between 1.0 kV and 4.0, five peaks for anion resin between 2.0 kV and 4.0 and six peaks for cation resin between 1.0 kV and 4.0. The EDS spectra also showed the presence of other major elements found in zeolite (Na, Mg, Al, Si, Ca, K, Ti), sand (C, Fe, Al, Si), fibreglass (Al, Si, Ca), anion resin (C, N, O, Al, Cl) and cation resin (C, O, Al, S). The carbon and oxygen peaks of the EDS analyses could be due to the carbon tape used for SEM stub preparation.

**Figure 3:**
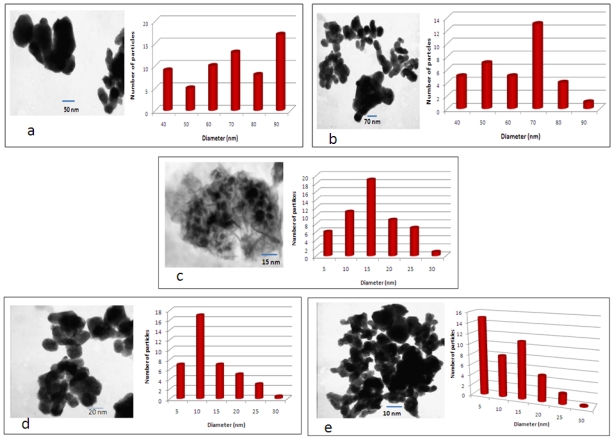
TEM image and particle size distribution of (**a**) zeolite, (**b**) sand, (**c**) fibreglass, (**d**) anion resin, and (**e**) cation resin coated with silver nanoparticles.

Figure 3 shows the results of the TEM image and particle size distribution histogram of silver nanoparticle substrates (zeolite, sand, fibreglass, anion and cation resin beads). The morphology of silver nanoparticles deposited onto substrates exhibited spherical-shaped particles that aggregate to each other. According to the particle-size distribution histogram, the silver nanoparticles have shown a majority particle sizes distributed from 40 to 90 nm for zeolite and sand, and 5 to 30 nm for fibreglass and resin beads.

**Figure 4:**
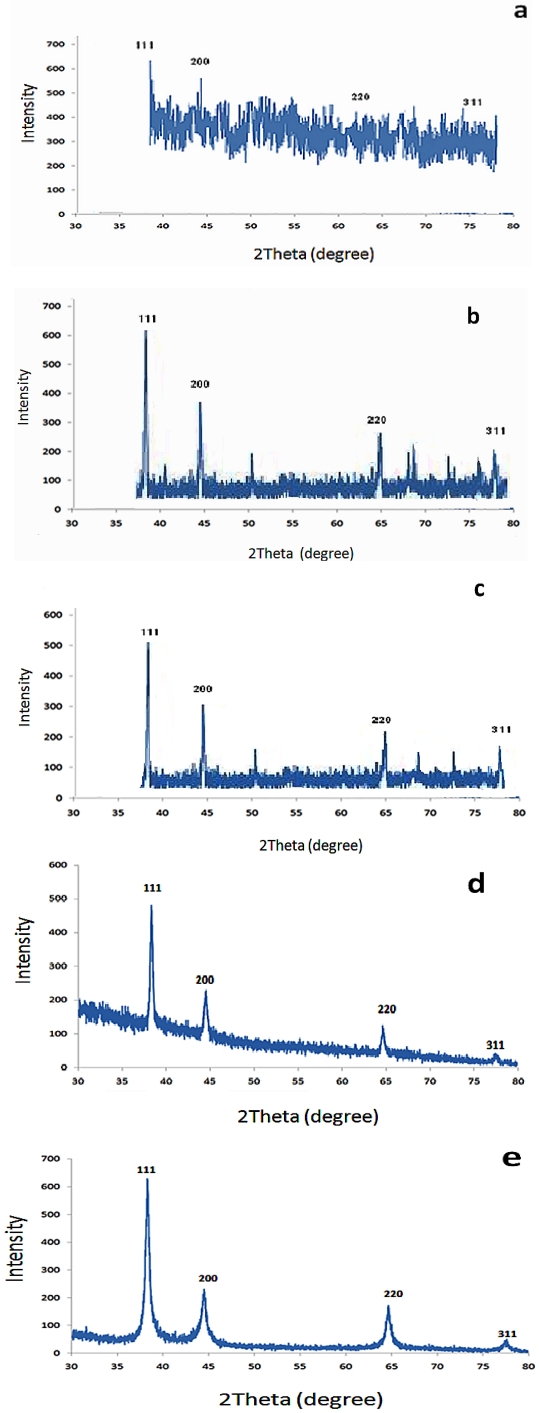
XRD patterns of the silver nanoparticles coated on (**a**) zeolite, (**b**) sand, (**c**) fiberglass, (**d**) anion resin, and (**e**) cation resin.

The diffraction pattern of the synthesised silver nanoparticle substrates shown in Figure 4 matched the face-centered cubic (fcc) structure of silver observed at 2θ angle 37.7°, 44.0°, 64.2° and 77.1°. These corresponded to the four diffraction peaks above 30° (111), (200), (220) and (311) crystal planes, respectively, clearly indicating that the silver nanoparticles are crystalline in nature. The XRD patterns were analysed to determine the average crystallite size obtained by classical Scherrer equation indicated in Section 2.3. The typical XRD pattern estimated average nanocrystallite sizes of 85 nm for zeolite, 80 nm for sand, 28 nm for fibreglass, 20 nm for anion resin and 13 nm for cation resin derived from full-width at half-maximum (FWHM) of peak corresponding to 111 plane with cubic shape. The zeolite substrate (Figure 4(a)) did not show any well-defined peaks which indicated an amorphous nature of the sample. All the substrates showed low-intensity peaks of silver at 2θ = 77.1°. As seen in Figure 4(a–c), in addition to the four peaks of the crystal plane, other unidentified peaks also appeared in the XRD pattern.

### 3.2. Characteristics of Test Water Sources Before Treatment

#### 3.2.1. Microbiological Quality of Test Water Sources

Table 1 illustrates the initial concentrations of *E. coli* spiked in sterile synthetic water and groundwater with targeted bacteria before treatment. The initial count of the suspension for *E. coli* spiked in synthetic water was approximately 6 log cfu/100 mL. Therefore, after mixing thoroughly, the average count of *E. coli* was found to be 7 log cfu/100 mL. Initial presumptive *E. coli* in groundwater had an average count of 3 log cfu/100 mL. *Salmonella typhimurium*, *S. dysenteriae* and *V. cholerae* were not originally detected in the test groundwater samples. Consequently, these water source samples were spiked with initial concentrations of approximately 3 log cfu/100 mL for each target organisms. However, after thoroughly mixing the spiked groundwater samples, the average counts for *S. typhimurium*, *S. dysenteriae* and *V. cholerae* as shown in Table 1 were approximately 3 log cfu/100 mL. 

**Table 1:** Microbiological quality of spiked water sources before treatment.

Bacterial concentrations (cfu/100 mL) before filtration					
		Targeted organisms			
Water sources	*Presumtive E. coli*		*S. typhimurium*	*S. dystenteriae*	*V. cholerae*
Synthetic water		3.21 × 10^7^	ND	ND	ND
Groundwater		3.15 × 10^3^	1.20 × 10^3^	2.71 × 10^3^	2.01 × 10^3^

ND-Not done.

#### 3.2.2. Physicochemical and Microbiological Quality of Groundwater Source

The characteristics of groundwater samples collected from a Delmas borehole (A7) are illustrated in Table 2. The average physicochemical values of groundwater were 7.22 for pH; 1.59 NTU for turbidity; 0.46 mg/L for fluorides; 1.59 mg/L for nitrates; 98.25 mg/L as Ca for calcium and 26.36 mg/L as Mg for magnesium, respectively. Regarding the microbiological quality, only presumptive *E. coli* (average count: 2.99 × 10^3^ cfu/100 mL) was present in groundwater samples. 

**Table 2:** Characteristics of groundwater sample.

Parameters	Units	Concentration	SANS 241
pH	-	7.22 ± 0.14	5–9.5
Turbidity	NTU	1.59 ± 0.11	<1
Fluorides	mg/L	0.46 ± 0.18	<1
Nitrates	mg/L as N	1.59 ± 0.02	<10
Calcium	mg/L as Ca	98.25 ± 1.12	<150
Magnesium	mg/L as Mg	26.36 ± 7.18	<70
*E. coli*	cfu/100 mL	2.99 × 10^3^	0

### 3.3. Performance of Combined Substrates-Silver Nanoparticles in Removing Pathogenic Bacteria from Synthetic Water

**Figure 5:**
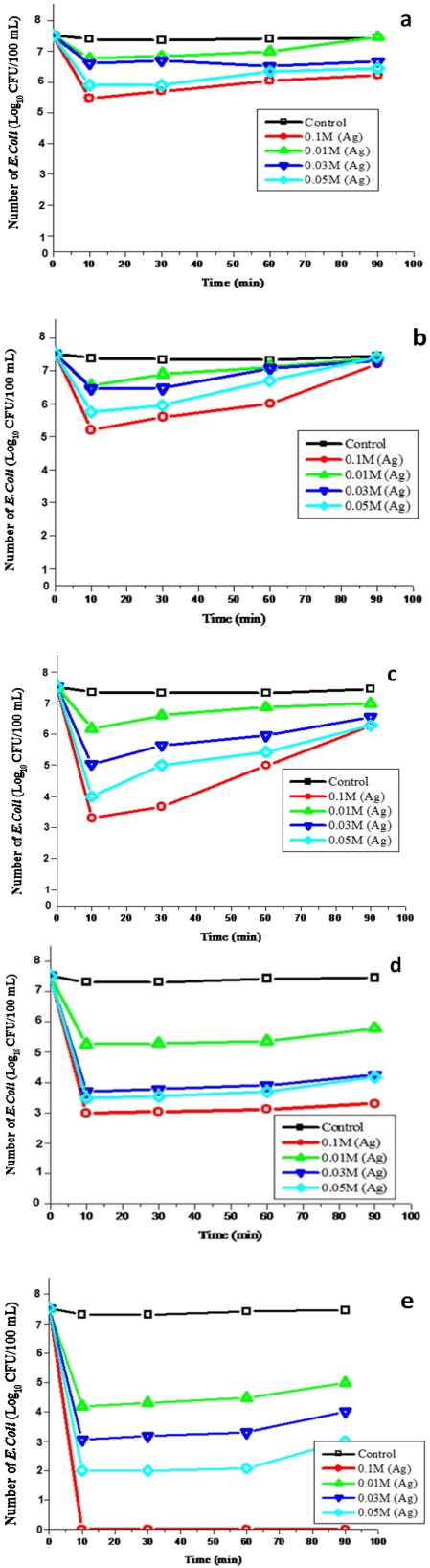
Antibacterial activity of various Ag nanoparticle-coated substrates against *E*. *coli* at different concentrations in synthetic water:(**a**) zeolite, (**b**) sand, (**c**) fibreglass, (**d**) anion resin, and (**e**) cation resin.

Figure 5 illustrates the effect of different concentrations of AgNO_3_ (0.01 mM, 0.03 mM, 0.05 mM and 0.1 mM) deposited on substrates for the deactivation of *E. coli*. As seen in Figure 5(a), the filter system with uncoated zeolite (control) did not have any antibactericidal effect on *E. coli*.However, Ag/zeolite nanoparticle filter systems with 0.01 mM and 0.03 mM concentrations showed a 0.5 log cfu/100 mL reduction of *E. coli*, while 0.05 mM and 0.1 mM concentrations showed a removal of l.0 log and 1.5 log cfu/100 mL for the first 30 min, respectively, and then an increase of bacterial counts resulting in a survival of more than 6 log cfu/100 mL occurred in the filtered water after 90 min trial. The results showed a significant difference in the reduction of the *E. coli* count with 0.03 mM, 0.05 mM and 0.1 mM concentrations (*P* < 0.05), as compared to the control.

As shown in Figure 5(b), the uncoated sand (control) filter system did not have any antibacterial effect on *E. coli*. Filter systems with Ag/sand nanoparticles reduced the *E. coli* count by 1.0 log cfu/100 mL with a 0.01 mM concentration in the first 10 min and with 0.03 mM in the first 30 min and there was an increase in the *E. coli* count of more than 7 log cfu/100 mL in the filtered water after 90 min trial. However, with 0.05 mM and 0.1 mM concentrations, a 2 log and 2.5 log cfu/100 mL reduction of the *E. coli* count was observed in the first 10 min, and subsequently the *E. coli* counts increased again in filtered water. The results showed a significant difference in the reduction of the *E. coli* count with filters containing 0.03 mM, 0.05 mM and 0.1 mM concentrations (*P* < 0.05), as compared to the control.

Figure 5(c) shows that no *E. coli* deactivation was observed with uncoated fibreglass (control). The filter systems with Ag/fibreglass nanoparticles indicated a reduction of 1.2 log cfu/100 mL *E. coli* in the first 10 min with a 0.01 mM concentration and 2.5 log cfu/100 mL with a 0.03 mM concentration during the same period. Thereafter, the growth of *E. coli* increased between 6 and 7 cfu/100 mL in the filtered water after 90 min trial. A high performance of the Ag/fibreglass nanoparticle filter system was observed when 0.05 mM and 0.1 mM concentrations were used. The results showed that 3.5 log cfu/ 100 mL *E. coli* was removed within the first 10 min with a 0.05 mM concentration and 4.3 log cfu/100 mL within the first 30 min with a 0.1 mM concentration, respectively. Thereafter, growth of *E .coli* in filtered water was noticed. There was a significant difference in the reduction of *E. coli* counts with these filter systems containing 0.03 mM, 0.05 mM and 0.1 mM concentrations (*P* < 0.05), as compared to the control.

As illustrated in Figure 5(d), the uncoated anion resin (control) filter system did not have any antibacterial effect against *E. coli*. Filter systems with Ag/anion nanoparticles reduced the *E. coli* count by 2.0 log cfu/100 mL with a 0.01 mM concentration in the first 10 min and there was an increase in the *E. coli* count in the filtered water. However, with 0.03 and 0.05 mM concentrations, a 4 log cfu/100 mL reduction of *E. coli* count was observed in the first 60 min, while with a 0.1 mM concentration a 4.5 log cfu/100 mL reduction of *E. coli* was also observed in the same period.Thereafter, the growth of *E. coli* increased between 3 and 5.6 cfu/100 mL in filtered water after 90 min trial. The results showed a significant difference in the reduction of the *E. coli* count with filters containing 0.03 mM, 0.05 mM and 0.1 mM concentrations (*P* < 0.05), as compared to the control.

The results of Ag/cation resin filter systems and uncoated cation resin (control) are shown in Figure 5(e). The results indicate that the uncoated resin filter did not have any antibacterial activity against *E. coli*. The filter systems with Ag/cation resin reduced *E. coli* by 3.5, 4.5 and 5.5 logs cfu/100 mL with 0.01 mM, 0.03 mM and 0.05 mM concentrations in the first 60 min of the filter run, respectively. Thereafter, a growth of between 2.5 and 4.8 cfu/100 mL *E. coli* in filtered water was noticed after 90 min trial. A complete removal (>7 log cfu/100 mL) of *E. coli* was achieved with 0.1 mM concentrations during the entire filter run (90 minutes) without a further regrowth in the treated water. There was a significant difference in the reduction of *E. coli* between filter systems containing 0.01 mM, 0.03 mM, 0.05 mM and 0.1 mM concentrations (*P* < 0.05), as compared to the control. However, the antibacterial efficiency of the substrates with different concentrations were found not be significantly different from one another (*P* > 0.05), except for the 0.1 mM concentration of Ag/cation resin (*P* < 0.05). 

### 3.4. Performance of Combined Substrates-Silver (0.1 mM) Nanoparticles in Removing Pathogenic Bacteria from Groundwater

Silver nanoparticle-coated substrates with 0.1 mM AgNO_3_ were selected for further investigation because of their performance efficiency. In general, the bactericidal activity of Ag nanoparticle substrates depended on the individual microorganism. For this reason, the test was carried out with Ag nanoparticle substrates against four bacterial strains by using groundwater containing presumptive *E. coli* with 3 log cfu/100 mL and seeded with 3 log cfu/mL of *S. typhimurium, S. dysenteriae* and *V. cholerae*.

**Figure 6:**
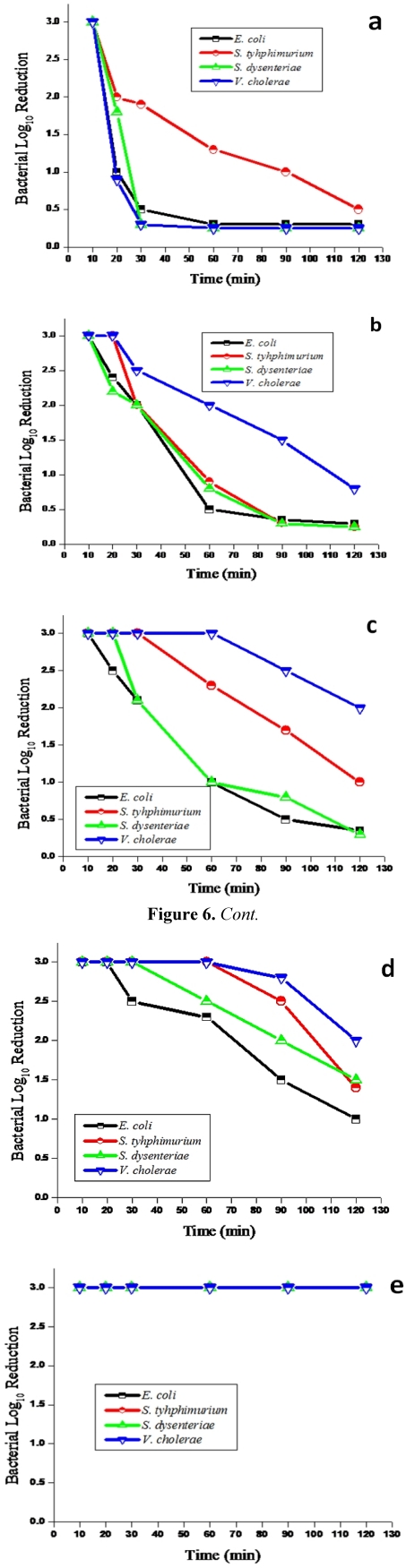
.Antibacterial activity of various Ag nanoparticle substrates at 0.01 mM concentration against *E. coli*, *S. typhimurium*, *S. dysenteriae* and *V. cholerae* in groundwater: (**a**) zeolite, (**b**) sand, (**c**) fibreglass, (**d**) anion resin, and (**e**) cation resin.

Figure 6 indicates the reduction of pathogenic bacteria in the treated water by five combined substrate-silver nanoparticle filter systems. The filter systems containing Ag/zeolite nanoparticles (Figure 6(a)) completely removed presumptive *E. coli*, *S. typhimurium, S. dysenteriae* and *V. cholerae* within the first 10 minutes of the filter run. Thereafter, these pathogenic bacteria reappeared in the treated water samples with counts ranging between 0.25 and 2 log removal. There was no significant difference (*P* > 0.05) in the regrowth of these pathogenic bacteria, except for the regrowth of *E. coli* and *S. typhimurium*, where a significant difference (*P* > 0.05) was observed with the Ag/zeolite nanoparticle filter system in the treated water.

The combined sand-silver nanoparticles filter system (Figure 6(b)) showed a complete removal (3 log) of presumptive *E. coli* and *S. dysenteriae* during the first 10 min of the filter run, while *S. typhimurium* and *V. cholerae* were completely removed within the first 20 min. Thereafter, *E. coli* and *S. dysenteriae* reappeared in the treated water samples for the rest of the study period and their counts ranged between 0.25 and 2.4 log cfu/100 mL, respectively. *Salmonella typhimurium* and *V. cholerae* reappeared in the treated water for the last 100 min of the filter run and their counts ranged between 0.25 and 2.5 log cfu/100 mL. There was no significant difference (*P* > 0.05) in the regrowth of these pathogenic bacteria with the Ag/sand nanoparticle filter system in the treated water.

The filter system with Ag/fibreglass nanoparticles (Figure 6(c)) completely removed (3 log cfu/100 mL) presumptive *E. coli* in the first 10 min, *S. dysenteriae* in the first 20 min, *S. typhimurium* in the first 30 min and *V. cholerae* in the first 60 min of the filter run. Subsequently, they reappeared in the treated water for the remaining time of the filter run and their counts ranged between 0.3 and 2.5 log cfu/100 mL. There was no significant difference (*P* > 0.05) in the regrowth of *E. coli*, *S. typhimurium* and *S. dysenteriae* with the Ag/fibreglass nanoparticle filter system, except for *E. coli* and *V. cholerae,* where there was a significant difference (*P* < 0.05) in the treated water.

The combined anion resin-silver nanoparticle filter system completely removed 3 log cfu/100 mL of presumptive *E. coli* in the first 20 min, *S. dysenteriae* in the first 30 min, and *S. typhimurium* and *V. cholerae* in the first 60 min of the filter run. Subsequently, these organisms progressively reappeared in the treated water with counts ranging between 1 and 2.8 log cfu/100 mL. There was no significant difference (*P* > 0.05) in the regrowth of these pathogenic bacteria with the Ag/anion resin nanoparticle filter system in the treated water.

The use of the cation resin-silver nanoparticle filter system (Figure 6(e)) resulted in a complete removal (3 log cfu/100 mL) of the target pathogenic bacteria and there was no bacterial regrowth during the entire 120 min of the filter run. Statistically, the performance of Ag/cation resin nanoparticles in removing all targeted pathogenic bacteria from groundwater was found to be significantly higher compared to the other filters when considering the phenomenon of bacterial regrowth that characterised the latter filters.

### 3.5. Elution of Silver Ions from Silver Nanoparticle Substrates

**Figure 7:**
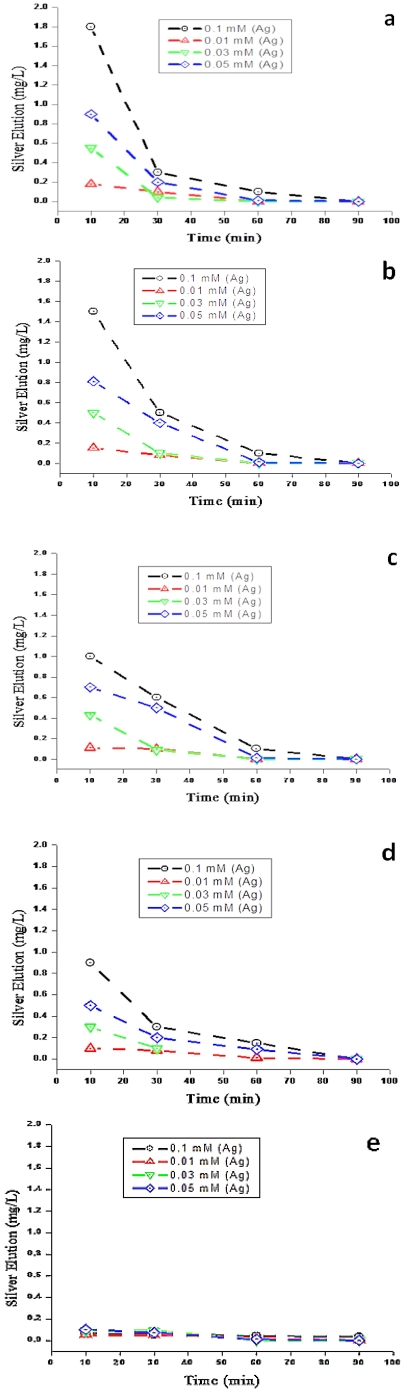
Amount of silver eluted from Ag nanoparticle-coated substrates in synthetic water: (**a**) zeolite, (**b**) sand, (**c**) fiberglass, (**d**) anion resin, and (**e**) cation resin.

Figure 7 shows the Ag^+^ ions eluted from filter materials coated with silver nanoparticles. A high concentration of silver was eluted from Ag/zeolite, Ag/sand, Ag/fiberglass and Ag/anion resin substrates within the first 10 minutes of the filter run. 

The content of silver released from these filters ranged between 1.0 and 1.8 mg/L for 0.1 mM Ag, between 0.7 and 0.8 mg/L for 0.05 mM Ag, between 0.5 and 0.6 mg/L for 0.03 mM Ag, and between 0.1 and 0.2 mg/L for 0.01 mM Ag. Consequently, after 90 min trial there was no silver ion detected in the filtered water from zeolite, sand fiberglass and anion resin substrates. However, the amount of the silver released from the Ag/cation resin was below 0.1 mg/L for all four the different concentrations investigated during the study period.

## 4. Discussion

The increasing demand for access to safe drinking water and the problems associated with centralised systems in developing countries have made decentralised systems vital for the development of new technologies to address these challenges, especially in scattered communities depending totally on groundwater supplies. Therefore, this study explored the use of nanosized silver impregnated onto cost-effective materials locally available in South Africa for possible use in drinking water disinfection. Using hydrothermal and chemical methods, it was revealed that silver nanoparticles were successfully deposited onto sand, zeolite, fibreglass, anion and cation resin substrates. However, in the case of the silver coatings, the antibacterial effect was found to reduce with time and the coatings had minimal antibacterial properties at 30 min. The ion elution studies (Figure 7) indicated that between 60 and 90% of silver loaded on the Ag/zeolite, Ag/sand, Ag/fibreglass and Ag/anion resin substrates eluted into the treated samples. Consequently the low level of Ag ions remaining in the surface substrates after 30 min elution is responsible for the decrease of bacterial removal at this time point. It was also discovered by [38] that silver eluted into water when using Ag-zeolite. The elution of silver from the substrates might be due to the weak attachment of silver nanoparticles to the surface substrates. High concentrations of silver ions eluted in water can be toxic to human cells and potentially cause adverse effects in the case of long-term implants. The amount of silver ions eluted from Ag/zeolite, Ag/sand, Ag/fibreglass and Ag/anion resin exceeded by far the recommended limit set by the World Health Organisation and US Environmental Protection Agency (USEPA), which is less than 0.10 mg/l in drinking water used for human consumption. The amount of silver ions eluted from Ag/cation resin complied with the WHO and USEPA limits [39,40]. Conversely, the high levels of ions (95%) still present in the Ag/cation resin surface after 30 min would explain the retention of the antimicrobial properties of that surface over the 90 min of the trial. Scanning electron microsope images revealed that the Ag nanoparticles on the substrates were predominantly small and spherical with EDS confirming the presences of silver peaks, as shown in Figure 2. The TEM images also revealed spherical-shaped particles that aggregated to each other on the silver nanoparticle substrates, which indicated a particle size distribution ranging between 5 and 90 nm. This result was in accordance with the results obtained from the XRD pattern lattice measurement corresponding to the (111) silver plane (Figure 4). The crystalline nature of Ag nanoparticles was confirmed by the XRD experimental study. The diffraction pattern observed from the XRD matches the face-centered cubic (fcc) structure of silver as described by previous investigators [41,42]. It was reported by [33] that the two broad reflection peaks corresponding to (111) and (200) planes indicate that the silver particles are nanocrystals with cubic symmetry. A similar observation was reported by [43]. According to these authors, both the nanoscale size and the presence of a (111) plane of Ag nanoparticles promote the biocidal property of *E. coli* [33,43]. 

The physicochemical analyses of the tested groundwater source were within the recommended limits of no risk for drinking except for turbidity which was above the SANS 241 limit [35]. High turbidity levels are associated with poor water quality and promote the survival of microorganisms [36]. Turbidity can also protect microorganisms from the effects of disinfection, stimulate the growth of bacteria and give rise to a significant disinfection demand [42]. 

*Escherichia coli* was found to be present at the highest concentration of 3 log cfu/100 mL in groundwater samples collected from Delmas, while the limit recommended by SANS 241 is 0 cfu/100 mL for drinking water that is meant for human consumption. Although *S. typhimurium*, *S. dysenteriae* and *V. cholerae* were not detected in the groundwater samples, these pathogenic bacteria were seeded into the groundwater samples at a concentration of approximately 3 log cfu/100 mL in order to determine the removal efficiency of the filters. In the first part of this study, the antibacterial efficiency of the combined substrates-silver nanoparticle filter systems were determined, using various concentrations of silver nitrate against *E. coli* as the test organism. The aim of this part of the study was to determine the concentration of silver that would have the most effective antibacterial property against the *E. coli*. The results indicated that all the filter systems containing uncoated substrates (Figure 5(a–e)) were unable to deactivate *E. coli* from synthetic water when compared to the combined substrates-silver nanoparticles filter systems. The bactericidal effect of silver nanoparticle substrates depended on the concentrations of the silver nitrate as well as on the type of substrates. The higher the concentration of Ag added to modify the substrates, the greater was the removal of *E. coli*. In analyses regarding the effect of silver nanoparticles in a size range of 5–90 nm, significant reductions in the *E. coli* population were noted when using filter materials coated with 0.01 mM, 0.03 mM, 0.05 mM and 0.1 mM silver concentrations as compared to the control (*P* < 0.05). The overall results indicated a significantly higher bactericidal efficiency with 0.1 mM AgNO_3_ (*P* < 0.05) compared to other silver concentrations, despite the phenomenon of bacterial regrowth that resulted in a progressive increase of bacterial counts in the treated water during the subsequent operation of all the filters, except for the Ag/cation resin filter (Figure 5(a–e)). This filter showed the best performance, which resulted in the complete removal of *E. coli* from synthetic water without the occurrence of bacterial regrowth when the cation resin was loaded with 0.1 mM silver (Figure 5(e)). The particle sizes of silver ranging between 1 and 100 nm have been reported to have an effect on the antibacterial properties of nanoparticles [14]. Silver nanoparticles cause irreversible damage to the cellular membrane [18,43,44], which enables the accumulation of nanoparticles in the cytoplasm. The action of silver nanoparticles is due to this damage and not to its toxicity [45]. Previous investigators have pointed out that Ag nanoparticles bind to the outer membrane of *E. coli,* causing the inhibition of active transport, dehydrogenase and periplasmic enzyme activity and eventually the inhibition of RNA, DNA and a decrease in the cell permeability, which finally results in cell lysis [18,45,46,47]. While microorganisms carry a negative charge, the Ag ions carry a positive charge, which is crucial for its antimicrobial activity through the electrostatic attraction between the negatively charged cell membrane of microorganisms and positively charged nanoparticles [48,49,50]. [51,52] indicated that the higher the concentration, the better the antibacterial activity will be. The percentage removal of *E. coli* from synthetic water was also in accordance with findings by previous investigations using a silver-coated ceramic water filter and silver-coated cylindrical polypropylene filters [53,54]. 

In the second part of the study, the antibacterial activities of combined substrates-silver nanoparticle filter systems prepared from 0.1 mM AgNO_3_ were investigated against *E. coli*, *S. typhimurium*, *S. dysenteriae* and *V. cholerae* found or spiked in groundwater samples. The results of this part of the study also showed a decrease in bacterial concentrations from groundwater samples by all filters (Figure 6). While the regrowth of targeted pathogenic bacteria occurred in water treated by Ag/zeolite, Ag/sand, Ag/fibreglass and Ag/anion resin, this phenomenon did not occur in drinking water treated with the Ag/cation resin nanoparticle filter system. This filter produced drinking water that complied with the limit of *E. coli* 0 cfu /100 mL as set by the South African guidelines [35,36]. The silver cation resin nanoparticle filter system achieved a 100% removal of all the targeted pathogenic bacteria during the entire 120 min of the filter operation. This performance of the Ag/cation resin nanoparticle filter system, namely removing 100% *E. coli*, was also reported by other researchers who used silver nanoparticle filters [55,56]. The results achieved in the first 10 min of the filter operation with a Ag/fibreglass nanoparticle filter system in removing *E. coli* confirmed those reported by [23] when these authors used a similar filter system for the purification of drinking water. Taking into account the performance of resin-silver nanoparticle filters in removing pathogenic bacteria and their level of silver ion elution in the drinking water, this suggests the use of this type of filter system as an alternative decentralisation technology for the production of safe drinking water for communities depending on groundwater supplies. 

## 5. Conclusions and Recommendations

Silver nanoparticle-coated substrates were prepared successfully by the chemical reduction and hydrothermal synthesis method. Detailed characterisation of the nanoparticles was carried out using SEM, EDS, TEM, Particle Size Distribution and XRD analyses, which confirmed the presences of silver loading on the substrates. The performance of the substrates as antibacterial water filter system was checked and no bacterium was detected in the output water when the Ag/cation resin substrate was used as a filter system. Low bacterial removal by Ag/zeolite, Ag/sand, Ag/fibrelass and Ag/anion resin filter systems was observed, which led to the conclusion that these systems are not ideal systems for the disinfection of drinking water. Consequently, a combined cation-resin silver nanoparticle system is the sole drinking-water purification system suggested by this study. This technology can offer complete anti-microbial solutions to rural communities. Further studies will be conducted on the lifespan of the combined cation-resin silver nanoparticle filter system and the communities will be informed of the period for which they can use this filter system
